# Impact of dual sensory impairment on cognition in older Chinese adults: a moderated chain-mediated effect

**DOI:** 10.3389/fpubh.2025.1542789

**Published:** 2025-04-03

**Authors:** Qingyun Mao, Heting Liang, Xiaoli Yuan, Zhixia Jiang, Rujun Hu, Yumeng Zhang, Shuang Li, Xiaoling Yang

**Affiliations:** ^1^Department of Nursing, Affiliated Hospital of Zunyi Medical University, Zunyi, Guizhou, China; ^2^Faculty of Nursing, Zunyi Medical University, Zunyi, Guizhou, China; ^3^College Office, Guizhou Nursing Vocational College, Guiyang, Guizhou, China

**Keywords:** dual sensory impairment, cognitive ability, anxiety, depression, frailty

## Abstract

**Objective:**

Although sensory impairment has been identified as a risk factor for cognitive decline, little is known about the underlying mechanisms that connect dual sensory impairment to cognitive ability. This research used a moderated chain-mediated model to investigate the underlying mechanisms behind the association between dual sensory impairment and cognitive ability.

**Methods:**

People aged 60 years and older from seven medical institutions, three communities, and five nursing homes in Zunyi city, Guizhou Province, were selected for the study from October 2022 to September 2023 via convenience sampling. Data on demographic characteristics, self-reported hearing and vision loss, and Self-Rating Anxiety Scale (SAS), 15-item Geriatric Depression Scale (GAD-15), Frailty Scale (FRAIL), and Mini-Mental State Examination (MMSE) scores were collected. A moderated chain mediator was used to analyze the underlying mechanisms and pathways of the relationships among anxiety, depression, and cognitive ability in individuals with dual sensory impairment, as well as the moderating role of frailty in this connection.

**Results:**

A total of 7,021 older adults were included, 3,598 (51.25%) of whom were male, with a mean age of 72.01 ± 7.17 years. Dual sensory impairment had a significant direct effect on cognitive ability, with an effect size of −3.134, followed by anxiety and depression, which not only independently mediated the relationship between dual sensory impairment and cognitive ability but also jointly had a chain mediation effect, with mediation effect sizes of −0.766 and −0.182, respectively, and a chain mediation effect size of −0.257. In addition, the interaction effect of dual sensory impairment and frailty was significantly predictive of cognitive ability (effect value = −0.575, *p* < 0.001).

**Conclusion:**

The mechanisms of action between dual sensory impairment, anxiety, depression, cognitive performance, and frailty are shown in this study. This finding also implies that therapies for psychological issues, frailty, and sensory functioning in older adults can preserve their cognitive ability.

## 1 Introduction

The phenomenon of the global population aging is becoming increasingly serious. The World Health Organization statistics indicate that more than 2 billion people will be older than 60 years, accounting for 21.1% of the world’s population, and the number of people with dementia worldwide is expected to reach 152 million by 2050 (1). Age is the greatest risk factor for cognitive impairment (2). Studies have shown an increased risk of cognitive impairment in older adults, often involving declines in one or more cognitive domains that eventually lead to severe cognitive impairment and even dementia (3–5). Cognitive impairment in older adults may reduce the ability to perform instrumental activities of daily living, the ability to cope with chronic diseases and self-care ability (6, 7); affect quality of life; and is associated with depression, cardiovascular events, and mortality (8, 9). Dementia is incurable and has a high mortality rate (10); therefore, identifying modifiable risk factors associated with cognitive decline is important.

With increasing age or disease, the incidence of visual impairment, hearing impairment and dual sensory impairment (both visual impairment and hearing impairment) in older adults gradually increases, which increases the cognitive load of visual and hearing information processing and impairs appropriate cognitive processing (11), seriously affecting their health and quality of life (12, 13). Although studies (11, 14, 15) have confirmed the relationship between dual sensory impairment and cognitive ability, few studies have investigated the mechanism of action between them.

Anxiety and depression are the most common and ignored psychological health problems in older adults, and they are related to cognitive decline in this population (16). Pardhan et al. (17) reported that the proportions of people with dual sensory impairment and anxiety and depression symptoms were 19.7% and 29%, respectively, which were 2–3 times greater than those of people without sensory impairment. Consistent with a previous study, the results of another study (18) also showed that older adults with dual sensory impairment had a greater risk of anxiety and depression. Thus, do anxiety and depression mediate the relationship between dual sensory impairment and cognitive ability in older adults?

The number of frail older adults is gradually increasing. Frailty is a clinical syndrome characterized by declining resistance to disease due to a decline in body function and physiological reserve function (19). Tan et al. (20) suggested that sensory impairments are potential markers of frailty, as they share some common risk factors, such as cognitive impairment, depression, physical dysfunction and disability. Second, frailty and cognitive decline are prevalent in older adults, and most studies have reported a correlation between frailty and cognitive decline (21–23). Since frailty is reversible (24), improvements in frailty may ease the development of anxiety and cognitive decline in older adults. Therefore, this study explored the moderating role of frailty in the relationship between dual sensory impairment and cognitive ability.

**Figure 1:**
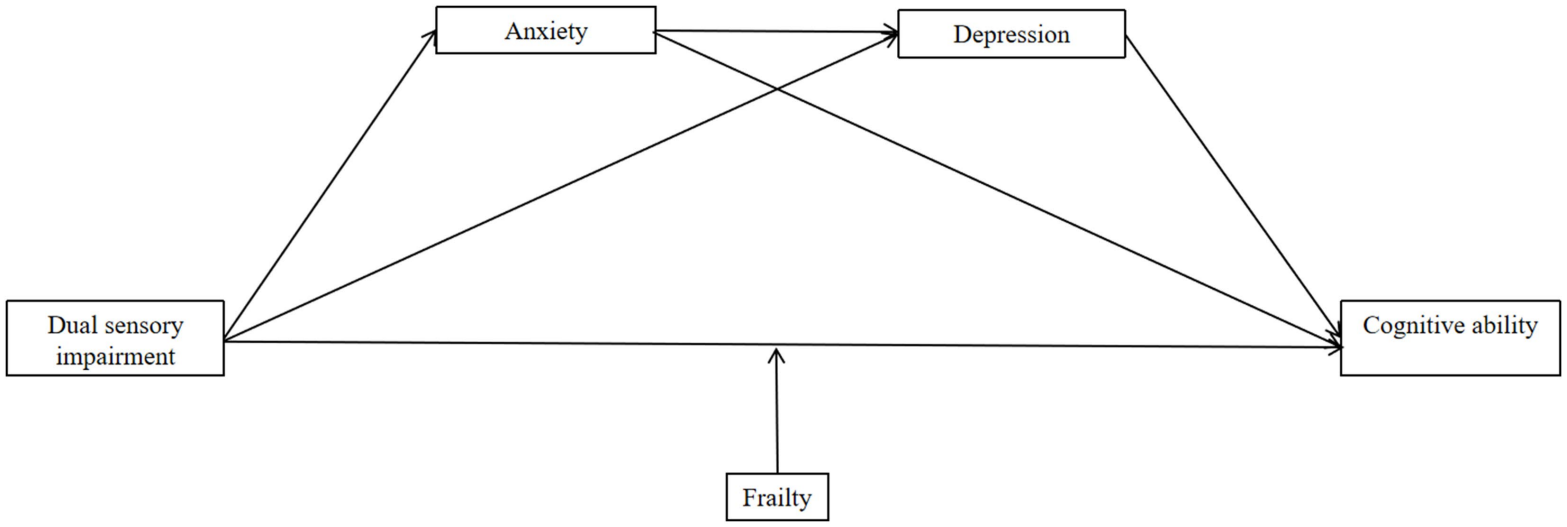
Hypothetical model for this study.

Moderated chain mediation analysis can delicately dissect and verify the strength and direction of the relationships between variables, revealing interaction mechanisms. In this study, we used this method to explore the relationships between dual sensory impairment, anxiety, depression, frailty and overall cognitive ability. This also further demonstrates how advanced statistical techniques can guide innovation and progress in public health research. In summary, through cross-sectional investigations and research, this study explores and examines the interaction mechanism and pathways of dual sensory impairment, anxiety, depression, frailty and cognitive ability through the moderated chain mediation and proposes the following hypotheses (Figure 1):

*Hypothesis 1:* Dual sensory impairment can directly affect cognitive ability.

*Hypothesis 2:* Anxiety and depression mediate the relationships between dual sensory impairment and cognitive ability. Additionally, dual sensory impairment can indirectly reduce cognitive ability through the chain mediation of anxiety and depression.

*Hypothesis 3:* Frailty moderates the relationship between dual sensory impairment and cognitive ability.

## 2 Methods

### 2.1 Participants

A convenience sampling method was used in this study, and data were collected from seven medical institutions, three communities, and five nursing homes in Zunyi city, Guizhou Province, from October 2022 to September 2023. The inclusion criteria for the participants were as follows: (1) ≥60 years of age, (2) no previous history of serious mental illness, clear consciousness, with normal communication ability, and (3) voluntary participation in this study after providing informed consent. The exclusion criteria were as follows: (1) had an acute or critical illness (including shock, respiratory failure, acute heart failure, acute myocardial infarction, or stroke) or were unable to cooperate with the investigators; and (2) had acute onset of a chronic disease or terminal disease. This study was reviewed by the Ethics Review Committee of the Affiliated Hospital of Zunyi Medical University (KLL2022-814) and conducted in accordance with the principles outlined in the Declaration of Helsinki. Each participant voluntarily agreed to participate in the study and provided informed consent.

### 2.2 Sample size

Cognitive ability is the study’s primary variable, research has shown that China’s rate of cognitive decline is approximately 28.6% (25). The study’s sample size is at least 1,962 instances, calculated by the formula’s 95% confidence interval (CI):


$$N=\frac{Z^{2}P\left(1-P\right)}{\delta^{2}}=\frac{1.96^{2}\times 0.286\times \left(1-0.286\right)}{0.02^{2}}\approx 1962$$

### 2.3 Demographic questionnaire

A demographic questionnaire was designed and used to collect data on the participants’ individual characteristics and health information, including sex, age, marital status, education level, living style, and residence type.

### 2.4 Self-reported sensory function

The participants were asked two questions to assess their vision and hearing. Question 1 was “Are you affected by poor vision when you engage in daily activities such as reading or watching TV?” The response options were set to “yes” and “no,” and participants who answered “yes” were considered to have vision difficulties. Question 2 was “Can you hear someone in the room speaking in a normal voice?” The response options were set to “yes” or “no,” and participants who answered “no” were considered to have hearing difficulties. The participants who reported both vision and hearing difficulties were described as having “dual sensory impairment.”

### 2.5 Self-Rating Anxiety Scale

Zung’s Self-Rating Anxiety Scale (SAS) (26), with a total of 20 items, was used in this study to determine the anxiety status of the participants. The response options were “never or occasionally,” “rarely,” “often,” and “always.” The total anxiety score was the sum of the scores of all the items multiplied by 1.25, and a score greater than 50 points indicated anxiety. The Cronbach’s *α* of this scale, which was used by Chinese scholars for older adults in nursing institutions, was 0.777 (27). The Cronbach’s α in this study was 0.837.

### 2.6 Short Form of the Geriatric Depression Scale

The Short Form of the Geriatric Depression Scale (GDS-15), which was developed by Sheikh et al. (28) and contains 15 items, was used to assess depression symptoms in older adult participants. The response options were “yes” and “no,” and the scores were “1” and “0,” respectively. The total score ranges from 0 to 15, and a score of 5 or more indicates depression (29, 30). The higher the score was, the greater the degree of depression. This scale has been shown to have good reliability and validity for the older adults in China (31). In this study, the Cronbach’s *α* was 0.715.

### 2.7 Mini-Mental State Examination

The Mini-Mental State Examination (MMSE) was used to evaluate the participants’ cognitive abilities. It was created by Folstein et al. (32), and modified by Zhang et al. (33) according to Chinese culture. Eight domains of cognitive ability—orientation, immediate memory, attention and calculation, delayed recall, and language—are evaluated through a total of 30 questions. Each right response earns one point, and the final score goes from 0 to 30, where higher scores denote superior cognitive ability. The MMSE has good reliability and validity and is suitable for Chinese older adult (34). In this study, the Cronbach’s *α* was 0.916.

### 2.8 FRAIL

The scale was proposed by Morely et al. (35) in 2008 by the International Society for Geriatric Nutrition and adapted for the Chinese population in 2018 by Wei-Yin et al. (36). It is a tool suitable for the clinical screening of older adult patients and consists of five main items: fatigue, increase in resistance/reduced endurance, decrease in free mobility, disease conditions, and weight loss. Each item is scored out of 1, with “yes” being scored out of 1 and “no” being scored out of 0. The total score ranges from 0 to 5, with a score of greater than or equal to 3 being considered frail. The Cronbach’s *α* for this study was 0.713.

### 2.9 Data collection

The questionnaires were reviewed for completeness of content and retrieved in a timely manner at the end of the survey to ensure the accuracy of the survey results. The questionnaires completed on the day of the survey were uniformly checked. All the evaluators were uniformly trained and qualified. The evaluator and the manager of the research site explained the purpose of the study to the participants in advance and obtained participant consent. Before the assessment, the purpose and precautions of the study were explained to the participants. After the participant provided consent and signed the informed consent form, the assessor asked the participants questions in a “face-to-face” manner in accordance with the unified guidance language and completed the questionnaire with the participants’ answers. The completed questionnaires will be checked on site to ensure completeness, accuracy and consistency.

### 2.10 Statistical analysis

SPSS 29.0 (IBM Inc., Armonk, NY, United States) was used for the data analysis. The quantitative data are expressed as the means ± standard deviations, and the qualitative data are expressed as percentages. The Kolmogorov–Smirnov test was used to determine whether the major variables were normally distributed. Spearman’s correlation was used to assess the correlations between dual sensory impairment and anxiety, depression, cognitive ability and frailty. The significance level was set at *p* < 0.05 (two-tailed). Mplus 8.3 (Los Angeles, CA, United States) was used to calculate the mediation parameters for the total, direct, and indirect effects and the moderating effects of frailty. The bootstrap sample number was set to 5,000, and a 95% confidence interval was used; if it did not contain 0, it indicated statistical significance.

## 3 Results

### 3.1 Description of demographic characteristics, the presence of dual sensory impairment, anxiety, depression, cognitive ability, and frailty

A total of 7,189 questionnaires were collected during the investigation, and after 168 invalid questionnaires were excluded, 7,021 older adults were ultimately included in this study, for an effective recovery rate of 97.66%. Among them, 4,122 participants (58.7%) were recruited from medical institutions, while 2,899 participants (41.3%) were recruited from communities and nursing homes. The descriptive analysis revealed that the average age of the participants in this study was 72.01 ± 7.17 years. There were 3,598 males (51.25%) and 3,265 (46.50%) older adult people with an education level below primary school as shown in Table 1.

**Table 1:** General demographic characteristics; the presence of dual sensory impairment; and anxiety, depression, and cognition scores (*N* = 7,021 people).

Variable		*n* (%)	M ± SD
Age (years)			72.01 ± 7.17
Sex	Male	3,598 (51.25)	—
	Female	3,423 (48.75)	—
Education level	Below primary school	3,265 (46.50)	—
	Primary	1,587 (22.60)	—
	Junior high school and above	2,169 (30.90)	—
Marital status	Married	6,133 (87.35)	—
	Single	888 (12.65)	—
Living style	Lives with others	6,563 (93.48)	—
	Lives alone	458 (6.52)	—
Residence type	Village	3,922 (55.86)	—
	Urban	3,099 (44.14)	—
Dual sensory impairment	No	6,524 (92.92)	—
	Yes	497 (7.08)	—
Anxiety		2,761 (39.3)	46.99 ± 10.17
Depression		1,927 (27.4)	3.82 ± 2.74
Cognitive ability		—	22.90 ± 6.26
Frailty		1,078 (15.4%)	0.92 + 1.32

^**^p < 0.001.

### 3.2 Correlation analysis among major variables

The matrix of correlation coefficients of the main variables of this study is shown in Table 2. Cognitive ability was significantly negatively correlated with dual sensory impairment, anxiety, depression, and frailty, followed by a significant positive correlation between dual sensory impairment and anxiety, depression, and frailty, in addition to a significant positive correlation between anxiety, depression, and frailty.

**Table 2:** Association of dual sensory impairment with anxiety, depression, cognitive ability, and frailty (*N* = 7,021).

	Dual sensory impairment	Anxiety	Depression	Cognitive ability	Frailty
Dual sensory impairment	1				
Anxiety	0.145^**^	1			
Depression	0.162^**^	0.457^**^	1		
Cognitive ability	−0.168^**^	−0.207^**^	−0.245^**^	1	
Frailty	0.176^**^	0.390^**^	0.396^**^	−0.261^**^	1

^**^p < 0.001.

### 3.3 Mediation of anxiety and depression

Mplus 8.3 (Los Angeles, CA, United States) was used to test the chain mediation effect. The results (see Table 3) revealed that dual sensory impairment significantly negatively predicted cognitive ability (*β* = −4.339, *p* < 0.001). After adding the mediator variable, dual sensory impairment still significantly negatively predicted cognitive ability (*β* = −3.134, *p* < 0.001). Moreover, dual sensory impairment was a significant positive predictor of anxiety and depression (β = 2.033, *p* < 0.001; β = 2.605, *p* < 0.001). Additionally, anxiety was a significant positive predictor of depression (β = 2.033, p < 0.001; β = 2.605, *p* < 0.001). had a significant positive predictive effect (β = 1.813, *p* < 0.001). These results suggest that anxiety and depression have significant single and chain mediating effects on the relationship between dual sensory impairment and cognitive ability, and hypothesis 2 is valid. Moreover, the direct effect of dual sensory impairment on cognitive ability as well as the mediating effects of anxiety and depression have bootstrap 95% confidence intervals of 0, suggesting that dual sensory impairment directly predicts cognitive ability through the independent mediating effects of anxiety and depression as well as a common chain of mediating effects to achieve prediction. The direct (−3.134) and mediating (−1.205) effects accounted for 72.23% and 27.77% of the total effect (−4.339), respectively.

**Table 3:** Results of the mediation analysis of anxiety and depression.

	Effect value	Boot SE	LLCI	ULCI	Relative effect value
Total effect	−4.339^**^	0.314	−4.967	−3.740	100%
Direct effect	−3.134^**^	0.303	−3.735	−2.553	72.23%
Indirect effect	−1.205^**^	0.105	−1.418	−1.000	27.77%
Ind1	−0.766^**^	0.087	−0.942	−0.599	17.65%
Ind2	−0.182^**^	0.034	−0.259	−0.123	4.19%
Ind3	−0.257^**^	0.035	−0.332	−0.195	5.92%

^**^p < 0.001. Boot SE, bootstrap standard error; LLCI, Lower Limit 95% Confidence interval; ULCI, Upper Limit 95% Confidence interval. Ind1 = Dual sensory impairment → Anxiety → Cognitive ability.

### 3.4 Moderating effect of frailty

**Figure 2:**
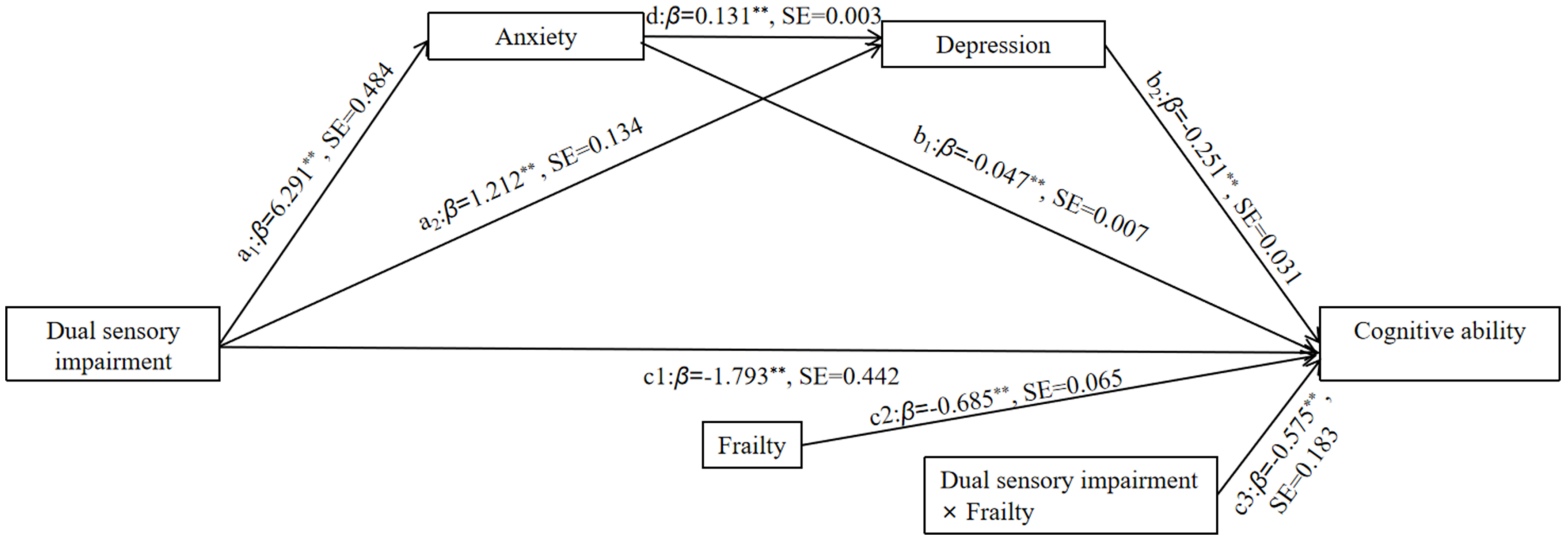
Path diagram for a moderated chain mediation model. ^**^p < 0.01 (two-tailed), a1, b1, c1, c2, and c3 all indicate *β* weights; SE, Standard error.

When frailty was included as a moderating variable in the model, the interaction term between dual sensory impairment and frailty was significant in predicting cognitive ability (*β* = −0.575, *p* < 0.001), suggesting that frailty has a moderating effect on the direct prediction of cognitive ability (Figure 2). Using plus or minus one standard deviation to categorize older adults into high and low groups to clarify trends in the moderating effect of frailty, the results indicated that for older adults with high levels of frailty (M + 1 SD), dual sensory impairment was a significant negative predictor of cognitive ability (*β* = −3.080, *p* < 0.001). For older adults with low levels of frailty (M − 1 SD), dual sensory impairment also significantly negatively predicted cognitive ability, but with a smaller effect (β = −1.561, *p* < 0.001). These findings suggest that the predictive effect of dual sensory impairment on cognitive ability changes with increasing levels of frailty (Table 4).

**Table 4:** Direct effects of different frailty levels.

	Effect value	Boot SE	LLCI	ULCI
High-level frailty	−1.561^**^	0.500	−2.571	−0.627
Moderate frailty	−2.321^**^	0.336	−3.011	−1.676
Low-level frailty	−3.080^**^	0.307	−3.686	−3.686

^**^p < 0.001. LLCI, Lower Limit 95% Confidence interval; ULCI, Upper Limit 95% Confidence interval.

## 4 Discussion

The study used moderated chain mediators to investigate the mechanisms and path sizes of anxiety and depression in the association between dual sensory impairment and cognitive ability and the moderating role of frailty in the association between dual sensory impairment and cognitive ability. Both sensory impairment, anxiety, depression, and frailty seriously affect the quality of life of older adults, and their impact on cognitive ability in the context of population aging is of concern. The results suggest that cognitive ability in older adults with dual sensory impairment is significantly predicted by both single mediation of anxiety and/or depression and chain mediation of anxiety and depression. Second, different levels of frailty moderated the direct predictive effect of dual sensory impairment on cognitive ability. Therefore, it is important to improve sensory function and frailty in older adults to curb their negative emotions and thus effectively slow cognitive decline.

### 4.1 Dual sensory impairment significantly negatively predicts cognitive ability

This study revealed that dual sensory impairment have significant direct negative predictive ability for cognitive ability in older adults, which is consistent with the results of previous studies (14, 15). Vision and hearing account for approximately 93% of age-related cognitive variation (37). The decrease in dual sensory function further complicates the relationship between vision and cognition and between hearing and cognition (14). Older adults with hearing and vision impairment require more cognitive resources to support hearing and vision functions, resulting in increased cognitive load, which results in fewer cognitive resources allocated to higher-order memory processes and accelerates cognitive decline (38). Regarding the biological mechanism, first, the “sensory deprivation” hypothesis suggests that long-term reduced auditory neuronal input due to hearing impairment leads to neuronal atrophy and cognitive decline (39). Additionally, the relationship between cognition and vision may be the reason that they share a common pathophysiological mechanism, and both involve hyperphosphorylated tau protein and amyloid-*β* protein, which are considered neurotoxic substances (40).

### 4.2 Anxiety and depression mediate the relationship between dual sensory impairment and cognitive ability

This study showed that anxiety and depression partially mediated the relationships between dual sensory impairment and cognitive ability. That is, older adults with dual sensory impairment are more likely to develop anxiety and/or depression, further affecting their cognitive ability. The limitations imposed by sensory impairment restrict social interactions and familial engagement for older individuals. Regular communication plays a crucial role in preventing depression in older adults (41). Lam et al. (42) further posited that interpersonal interaction reduces feelings of loneliness and enhances social participation among older adults, thereby positively influencing their mental well-being. Nevertheless, older adults with dual sensory impairment face certain constraints in this regard and are more likely to experience a sense of helplessness that naturally impacts their psychological state. Nyberg et al. (43) demonstrated that anxiety was significantly associated with impaired cognitive function, particularly in the domains of attention and executive functioning. Specifically, anxiety was found to negatively affect complex attention and processing speed, thereby disrupting cognitive processes. Moreover, anxiety was shown to lead to slower processing speed, which in turn exacerbated anxiety symptoms and further impaired cognitive function (44). On the other hand, anxiety can have a direct effect on the brain through the hypothalamic–pituitary axis, leading to cognitive decline. In addition, an increase in anxiety can exacerbate the effect of Aβ on cognitive decline in patients with preclinical dementia, resulting in rapid decreases in overall cognition, verbal memory, language and executive function (45). The structural and functional damage caused by depression in the brain is closely related to memory learning and executive functions (46); for example, a reduction in hippocampal volume in older adult depressed patients was associated with cognitive decline (47).

### 4.3 Anxiety and depression play a chain mediating role in the effects of dual sensory impairment on cognitive ability in older adult individuals

Our results show that anxiety and depression have a chain mediating effect on the relationship between dual sensory impairment and cognitive ability, and the chain mediating effect accounts for 5.92% of the total effect. In other words, older adults with dual sensory impairment are more likely to suffer anxiety, which further induces depression, thereby negatively affecting their cognitive ability. Anxiety and depression are prevalent among older adults and have multifaceted negative effects on their cognitive abilities. Parra-Díaz et al. (48) found that anxiety and depression symptoms were significantly associated with impaired attention, which may manifest as difficulties in concentrating, completing tasks, or maintaining conversations. Additionally, these symptoms can affect the functioning of the prefrontal cortex, thereby impacting decision-making, planning, and working memory. Anxiety and depression not only affect memory and attention but also have negative impacts on executive function, language ability, and calculation skills. These effects are likely related to underlying neurobiological mechanisms, such as changes in brain structure and imbalances in neurotransmitters (49). The interdependence between anxiety and depression has long been proposed and described. Anxiety and depression are highly comorbid, with an estimated incidence of as high as 60% or more (50); moreover, they have similar psychological and biological mechanisms, such as dysfunction in the serotonergic system (51). Additionally, a previous study showed that anxiety was accompanied by depressive symptoms and may be a precursor syndrome expected in the development of some forms of depression (52). Anxiety and depression are common influencing factors in physical, psychological and social aspects. For example, sleep disorders, physical health conditions and perceived pressure have a great impact on anxiety and depression, and social contact with family and friends also has an effect on anxiety and depression (53). Finally, individuals with symptoms of anxiety and depression often exhibit avoidant tendencies or are overwhelmed by emotional experiences, which further leads to depressed emotional states and increased worry, creating a vicious cycle (54). These factors may explain the interdependent relationship between depression and anxiety. Attention should be given to the deterioration of hearing and vision status and mental health problems in older adults, and targeted preventive measures should be taken to delay cognitive decline.

### 4.4 Moderating role of frailty

The study included frailty as a moderating variable in the model and revealed that although dual sensory impairment can directly affect cognitive performance, this effect may vary depending on the degree of frailty. The occurrence of frailty in older adults leads to a decline in physiological reserve capacity and an increase in vulnerability, while the decline in physical function further exacerbates frailty, creating a vicious cycle. Sensory impairment can increase the risk of functional disability in older adults, such as difficulties in performing activities of daily living (ADL) or instrumental activities of daily living (IADLs) (55). This, in turn, restricts physical activity levels and affects muscle mass, muscle strength, and function (56). Additionally, sensory impairment increases the risk of frailty, cognitive impairment, anxiety, and depression (57, 58). Furthermore, multiple studies have confirmed the association between frailty and cognitive function, suggesting that the link between frailty and cognitive decline may be mediated through inflammatory pathways. A study by Li et al. (59) revealed that inflammatory cell scores, such as white blood cell (WBC) counts, neutrophil (NE) counts, and neutrophil-to-lymphocyte ratios (NLRs), were significantly greater in the cognitively impaired group than in the normal group. Next, systemic inflammatory indices and cognitive decline were positively correlated. The frail state was also found to have significantly increased levels of the proinflammatory cytokines TNF-*α* and CRP, according to studies by Lai et al. (60) and Hammani et al. (61). These mediators may activate microglia in the brain, causing a malignant inflammatory cycle that damages crucial neurons and impairs cognitive ability. Therefore, frailty moderates the link between dual sensory impairment and cognitive ability, and therapies for frailty can moderate the effects of dual sensory impairment on cognitive ability.

This study leveraged a large sample to elucidate the mechanisms and pathways linking dual sensory impairment, anxiety, depression, cognitive function, and frailty. However, several limitations should be noted. First, this study is cross-sectional and cannot elucidate the causal relationships between dual sensory impairment, anxiety, depression, cognitive decline, and frailty. These relationships could be confirmed through prospective longitudinal studies. Second, the participants in this study were recruited from medical institutions, communities, and nursing homes, without analysis of cognitive function across different backgrounds of older adults. Future research should explore the physiological, psychological, and social dimensions of cognitive function in older adults from varied contexts. Lastly, this study focused only on the relationships between frailty, dual sensory impairment, anxiety, depression, and overall cognitive function, without including specific cognitive domains in the analysis. Future research could further investigate these domains and incorporate objective indicators, such as laboratory biochemical markers, as well as a broader range of modifiable risk factors, such as social and physiological factors, to expand the scope of potential clinical cognitive interventions.

## 5 Conclusion

In conclusion, the relationships between dual sensory impairment and cognitive ability are mediated by anxiety and depression, whereas frailty plays a moderating role in this relationship. In other words, mental health issues such as anxiety and/or depression are more common in older adults with multiple sensory impairments, and severe anxiety and/or depression can worsen cognitive loss. According to previous studies, preventing and preserving cognitive abilities in older adults requires improving early sensory and psychological assessments, frailty therapies, and customized interventions, such as the use of hearing aids and reading glasses, to reduce the degree of sensory impairment in older adults and reduce the occurrence of mental health problems, which are highly important for the prevention and protection of cognitive ability in older adults.

## Data Availability

The original contributions presented in the study are included in the article/supplementary material, further inquiries can be directed to the corresponding authors.

## References

[1] Chatterji S Byles J Cutler D Seeman T Verdes E. Health, functioning, and disability in older adults--present status and future implications. Lancet. (2015) 385:563–75. doi: 10.1016/s0140-6736(14)61462-8, PMID: 25468158 PMC4882096

[2] Dunne RA Aarsland D O'Brien JT Ballard C Banerjee S Fox NC. Mild cognitive impairment: the Manchester consensus. Age Ageing. (2021) 50:72–80. doi: 10.1093/ageing/afaa228, PMID: 33197937 PMC7793599

[3] Valech N Tort-Merino A Coll-Padrós N Olives J León M Rami L. Executive and language subjective cognitive decline complaints discriminate preclinical Alzheimer's disease from Normal aging. J Alzheimers Dis. (2018) 61:689–703. doi: 10.3233/jad-170627, PMID: 29254090

[4] Sánchez-Benavides G Grau-Rivera O Suárez-Calvet M Minguillon C Cacciaglia R Gramunt N. Brain and cognitive correlates of subjective cognitive decline-plus features in a population-based cohort. Alzheimers Res Ther. (2018) 10:123. doi: 10.1186/s13195-018-0449-9, PMID: 30572953 PMC6302483

[5] Chi SY Chua EF Kieschnick DW Rabin LA. Retrospective Metamemory monitoring of semantic memory in community-dwelling older adults with subjective cognitive decline and mild cognitive impairment. Neuropsychol Rehabil. (2022) 32:429–63. doi: 10.1080/09602011.2020.1831552, PMID: 33106082 PMC8071835

[6] Rong H Lai X Jing R Wang X Fang H Mahmoudi E. Association of Sensory Impairments with cognitive decline and depression among older adults in China. JAMA Netw Open. (2020) 3:e2014186. doi: 10.1001/jamanetworkopen.2020.14186, PMID: 32990739 PMC7525357

[7] Mueller-Schotte S Zuithoff NPA van der Schouw YT Schuurmans MJ Bleijenberg N. Trajectories of limitations in instrumental activities of daily living in frail older adults with vision, hearing, or dual sensory loss. J Gerontol A Biol Sci Med Sci. (2019) 74:936–42. doi: 10.1093/gerona/gly155, PMID: 29982391

[8] Jaeger J Berns S Uzelac S Davis-Conway S. Neurocognitive deficits and disability in major depressive disorder. Psychiatry Res. (2006) 145:39–48. doi: 10.1016/j.psychres.2005.11.011 17045658

[9] O'Donnell M Teo K Gao P Anderson C Sleight P Dans A. Cognitive impairment and risk of cardiovascular events and mortality. Eur Heart J. (2012) 33:1777–86. doi: 10.1093/eurheartj/ehs053 22551598

[10] Zhang D Zheng W Li K. The relationship between marital status and cognitive impairment in Chinese older adults: the multiple mediating effects of social support and depression. BMC Geriatr. (2024) 24:367. doi: 10.1186/s12877-024-04975-6, PMID: 38658842 PMC11040757

[11] Tomida K Lee S Bae S Harada K Katayama O Makino K. Association of Dual Sensory Impairment with cognitive decline in older adults. Dement Geriatr Cogn Disord. (2022) 51:322–30. doi: 10.1159/000525820, PMID: 35896063

[12] Liang H Jiang Z Yang X Li S Zhao X Dai Y. The interaction between instrumental activities of daily living and dual sensory function on cognition among the elderly in China: a cross-sectional survey. Ibrain. (2023) 9:281–9. doi: 10.1002/ibra.12124, PMID: 37786757 PMC10527793

[13] Huang AR Rebok GW Swenor BK Reed N Griswold M Zhu X. Concurrent hearing and vision impairment and 8-year memory decline in community-dwelling older adults. Alzheimers Dement. (2023) 19:2307–16. doi: 10.1002/alz.12887, PMID: 36462211 PMC10238672

[14] Dumassais S Pichora-Fuller MK Guthrie D Phillips NA Savundranayagam M Wittich W. Strategies used during the cognitive evaluation of older adults with dual sensory impairment: a scoping review. Age Ageing. (2024) 53:afae051. doi: 10.1093/ageing/afae051, PMID: 38506649 PMC10953621

[15] He W Li P Gao Y You J Chang J Qu X. Self-reported visual impairment and depression of middle-aged and older adults: the chain-mediating effects of internet use and social participation. Front Public Health. (2022) 10:957586. doi: 10.3389/fpubh.2022.957586, PMID: 36466466 PMC9714326

[16] Lü W Duan J Zhang W Yang W Yu W. Relationship between neuropsychiatric symptoms and cognitive functions in patients with cognitive impairment. Psychogeriatrics. (2021) 21:773–82. doi: 10.1111/psyg.12738, PMID: 34216181

[17] Pardhan S Smith L Bourne R Davis A Leveziel N Jacob L. Combined vision and hearing difficulties results in higher levels of depression and chronic anxiety: data from a large sample of Spanish adults. Front Psychol. (2020) 11:627980. doi: 10.3389/fpsyg.2020.627980, PMID: 33536989 PMC7848112

[18] Soto-Perez-de-Celis E Sun CL Tew WP Mohile SG Gajra A Klepin HD. Association between patient-reported hearing and visual impairments and functional, psychological, and cognitive status among older adults with Cancer. Cancer. (2018) 124:3249–56. doi: 10.1002/cncr.31540, PMID: 29797664 PMC6613937

[19] Kaushal N Langlois F Desjardins-Crépeau L Hagger MS Bherer L. Investigating dose-response effects of multimodal exercise programs on health-related quality of life in older adults. Clin Interv Aging. (2019) 14:209–17. doi: 10.2147/cia.S187534, PMID: 30774322 PMC6352872

[20] Tan BKJ Man REK Gan ATL Fenwick EK Varadaraj V Swenor BK. Is sensory loss an understudied risk factor for frailty? A systematic review and Meta-analysis. J Gerontol A Biol Sci Med Sci. (2020) 75:2461–70. doi: 10.1093/gerona/glaa171, PMID: 32735331

[21] Topcuoglu C Vardar Yagli N Aykan HH Ertugrul I Karagoz T Saglam M. Exploring frailty: muscle strength, functional capacity, activities of daily living and cognition in adult congenital heart disease. Disabil Rehabil. (2024):1–7. doi: 10.1080/09638288.2024.2417775, PMID: 39460676

[22] Lin X Nian Z Yang L Qing Z Zhenjun N Yanlin H. Prevalence and influencing factors of cognitive frailty among Chinese older adults: a systematic review and Meta-analysis. Int J Nurs Pract. (2024) 30:e13306. doi: 10.1111/ijn.13306 39448383

[23] Lorenzo-López L Cibeira N Hemadeh A López-López R Lema-Arranz C Maseda A. Association between cognitive reserve proxies and frailty phenotype: Data from UK biobank. GeroScience. (2024). doi: 10.1007/s11357-024-01382-y, PMID: 39397220 PMC11978585

[24] Kolle AT Lewis KB Lalonde M Backman C. Reversing frailty in older adults: a scoping review. BMC Geriatr. (2023) 23:751. doi: 10.1186/s12877-023-04309-y, PMID: 37978444 PMC10655301

[25] Jia X Wang Z Huang F Su C Du W Jiang H. A comparison of the Mini-mental state examination (Mmse) with the Montreal cognitive assessment (Moca) for mild cognitive impairment screening in Chinese middle-aged and older population: a cross-sectional study. BMC Psychiatry. (2021) 21:485. doi: 10.1186/s12888-021-03495-6, PMID: 34607584 PMC8489046

[26] Zung WW. A rating instrument for anxiety disorders. Psychosomatics. (1971) 12:371–9. doi: 10.1016/s0033-3182(71)71479-0, PMID: 5172928

[27] Huiyuan L Xueqi G Qiqun G Jiao Y Yifan G Tianshu S. Study on the status and influencing factors of sleep beliefs and attitudes of the elderly in Elderly care institutions. Modern Prev Med. (2023) 50:1637–42. doi: 10.20043/j.cnki.MPM.202209340

[28] Sheikh JI Yesavage JA. Geriatric Depression Scale (Gds) Recent Evidence and Development of a Shorter Version. Clin Gerontol. (1986) 5:165–73.

[29] Guerin JM Copersino ML Schretlen DJ. Clinical utility of the 15-item geriatric depression scale (Gds-15) for use with young and middle-aged adults. J Affect Disord. (2018) 241:59–62. doi: 10.1016/j.jad.2018.07.038, PMID: 30096593

[30] Asokan GV Awadhalla M Albalushi A Al-Tamji S Juma Z Alasfoor M. The magnitude and correlates of geriatric depression using geriatric depression scale (Gds-15)—a Bahrain perspective for the who 2017 campaign 'Depression—Let's talk. Perspect Public Health. (2019) 139:79–87. doi: 10.1177/1757913918787844, PMID: 29993323

[31] Mei J. Reliability and validity of Gds and Ghq short form for the aged. Chin J Psychiatry. (1999):40–2. doi: 10.3760/j:issn:1006-7884.1999.01.013

[32] Folstein MF Folstein SE McHugh PR. "Mini-mental state". A practical method for grading the cognitive state of patients for the clinician. J Psychiatr Res. (1975) 12:189–98. doi: 10.1016/0022-3956(75)90026-6, PMID: 1202204

[33] Wang Z Zhang M. Chinese version of the application of the Mini-mental state examination (Mmse). Shanghai Arch Psychiatry. (1989) 7:4.

[34] Xiong Y Yang X Yang J Xue W Zhou Q Qu F. Relationship between social support and cognitive function and mediation of anxiety and sleep in rural aged people in Guizhou. Modern Prev Med. (2022) 49:3717–22. doi: 10.20043/j.cnki.MPM.202204492

[35] Morley JE Malmstrom TK Miller DK. A simple frailty questionnaire (frail) predicts outcomes in middle aged African Americans. J Nutr Health Aging. (2012) 16:601–8. doi: 10.1007/s12603-012-0084-2, PMID: 22836700 PMC4515112

[36] Yin W Yanpei C Xiaoli Y Yan X. Reliability and validity of the Chinese version of fatigue, resistance, ambulation, illness, and loss for elder inpatients. Chin J Pract Nurs. (2018) 34:1526–30. doi: 10.3760/cma.j.issn.1672-7088.2018.20.002

[37] Baltes PB Lindenberger U. Emergence of a powerful connection between sensory and cognitive functions across the adult life span: a new window to the study of cognitive aging? Psychol Aging. (1997) 12:12–21. doi: 10.1037//0882-7974.12.1.12, PMID: 9100264

[38] Bikbov MM Kazakbaeva GM Rakhimova EM Rusakova IA Fakhretdinova AA Tuliakova AM. Concurrent vision and hearing impairment associated with cognitive dysfunction in a population aged 85+ years: the Ural very old study. BMJ Open. (2022) 12:e058464. doi: 10.1136/bmjopen-2021-058464, PMID: 35473730 PMC9045115

[39] Valentijn SA van Boxtel MP van Hooren SA Bosma H Beckers HJ Ponds RW. Change in sensory functioning predicts change in cognitive functioning: results from a 6-year follow-up in the Maastricht aging study. J Am Geriatr Soc. (2005) 53:374–80. doi: 10.1111/j.1532-5415.2005.53152.x, PMID: 15743277

[40] Jain S Aref AA. Senile dementia and Glaucoma: evidence for a common link. J Ophthal Vis Res. (2015) 10:178–83. doi: 10.4103/2008-322x.163766, PMID: 26425322 PMC4568617

[41] Nakagomi A Shiba K Kondo K Kawachi I. Can online communication prevent depression among older people? A longitudinal analysis. J Appl Gerontol. (2022) 41:167–75. doi: 10.1177/0733464820982147, PMID: 33356760

[42] Lam SSM Jivraj S Scholes S. Exploring the relationship between internet use and mental health among older adults in England: longitudinal observational study. J Med Internet Res. (2020) 22:e15683. doi: 10.2196/15683, PMID: 32718913 PMC7420689

[43] Nyberg J Henriksson M Wall A Vestberg T Westerlund M Walser M. Anxiety severity and cognitive function in primary care patients with anxiety disorder: a cross-sectional study. BMC Psychiatry. (2021) 21:617. doi: 10.1186/s12888-021-03618-z, PMID: 34886841 PMC8662874

[44] Vissicchio NA Altaras C Parker A Schneider S Portnoy JG Archetti R. Relationship between anxiety and cognition in multiple sclerosis: implications for treatment. Int J MS Care. (2019) 21:151–6. doi: 10.7224/1537-2073.2018-027, PMID: 31474807 PMC6709568

[45] Li XX Li Z. The impact of anxiety on the progression of mild cognitive impairment to dementia in Chinese and English data bases: a systematic review and Meta-analysis. Int J Geriatr Psychiatry. (2018) 33:131–40. doi: 10.1002/gps.4694, PMID: 28240415

[46] Yuan F Hui L Song Y Qiutong C Hongjie Y Mingwei L. Analysis of cognitive function status and its influencing factors among middle-aged and elderly people in China. J Public Health Prev Med. (2019) 30:75–8. doi: 10.3969/j.issn.1006-2483.2019.03.017

[47] Steffens DC Payne ME Greenberg DL Byrum CE Welsh-Bohmer KA Wagner HR. Hippocampal volume and incident dementia in geriatric depression. Am J Geriatr Psychiatry. (2002) 10:62–71. doi: 10.1097/00019442-200201000-00008, PMID: 11790636

[48] Parra-Díaz AB Aibar-Almazán A Martínez-Amat A Jiménez-García JD Álvarez-Salvago F Hita-Contreras F. Associations of sleep quality, anxiety, and depression with cognitive and executive functions among community-dwelling women aged ≥ 65 years: a cross-sectional study. Healthcare. (2021) 9:599. doi: 10.3390/healthcare9111599, PMID: 34828644 PMC8623846

[49] Castaneda AE Tuulio-Henriksson A Marttunen M Suvisaari J Lönnqvist J. A review on cognitive impairments in depressive and anxiety disorders with a focus on young adults. J Affect Disord. (2008) 106:1–27. doi: 10.1016/j.jad.2007.06.006, PMID: 17707915

[50] Kessler RC Sampson NA Berglund P Gruber MJ Al-Hamzawi A Andrade L. Anxious and non-anxious major depressive disorder in the World Health Organization world mental health surveys. Epidemiol Psychiatr Sci. (2015) 24:210–26. doi: 10.1017/s2045796015000189, PMID: 25720357 PMC5129607

[51] Bui E Fava M. From depression to anxiety, and Back. Acta Psychiatr Scand. (2017) 136:341–2. doi: 10.1111/acps.12801, PMID: 28865404

[52] Liu Y Feng Q Guo K. Physical activity and depression of Chinese college students: chain mediating role of rumination and anxiety. Front Psychol. (2023) 14:1190836. doi: 10.3389/fpsyg.2023.1190836, PMID: 37583607 PMC10423818

[53] Shin H Park C. Mastery is central: an examination of complex interrelationships between physical health, stress and adaptive cognition, and social connection with depression and anxiety symptoms. Front Psych. (2024) 15:1401142. doi: 10.3389/fpsyt.2024.1401142, PMID: 38751422 PMC11094708

[54] Berenbaum H Bredemeier K Thompson RJ Boden MT. Worry, Anhedonic depression, and emotional styles. Cogn Ther Res. (2012) 36:72–80. doi: 10.1007/s10608-010-9329-8, PMID: 40123990

[55] Davidson JGS Guthrie DM. Older adults with a combination of vision and hearing impairment experience higher rates of cognitive impairment, functional dependence, and worse outcomes across a set of quality indicators. J Aging Health. (2019) 31:85–108. doi: 10.1177/0898264317723407, PMID: 28805100

[56] Virtuoso Júnior JS Roza LB Tribess S Meneguci J Mendes EL Pegorari MS. Time spent sitting is associated with changes in biomarkers of frailty in hospitalized older adults: a cross sectional study. Front Physiol. (2017) 8:505. doi: 10.3389/fphys.2017.00505, PMID: 28824439 PMC5534441

[57] Coelho L Hauck K McKenzie K Copeland JL Kan IP Gibb RL. The association between sedentary behavior and cognitive ability in older adults. Aging Clin Exp Res. (2020) 32:2339–47. doi: 10.1007/s40520-019-01460-8, PMID: 31898168

[58] Giurgiu M Plotnikoff RC Nigg CR Koch ED Ebner-Priemer UW Reichert M. Momentary mood predicts upcoming real-life sedentary behavior. Scand J Med Sci Sports. (2020) 30:1276–86. doi: 10.1111/sms.13652, PMID: 32150774

[59] Li W Li S Shang Y Zhuang W Yan G Chen Z. Associations between dietary and blood inflammatory indices and their effects on cognitive function in elderly Americans. Front Neurosci. (2023) 17:1117056. doi: 10.3389/fnins.2023.1117056, PMID: 36895419 PMC9989299

[60] Lai HY Chang HT Lee YL Hwang SJ. Association between inflammatory markers and frailty in institutionalized older men. Maturitas. (2014) 79:329–33. doi: 10.1016/j.maturitas.2014.07.014, PMID: 25132319

[61] Hammami S Ghzaiel I Hammouda S Sakly N Hammami M Zarrouk A. Evaluation of pro-inflammatory cytokines in frail Tunisian older adults. PLoS One. (2020) 15:e0242152. doi: 10.1371/journal.pone.0242152, PMID: 33166358 PMC7652286

